# Driving hospital transformation with SLMTA in a regional hospital in Cameroon

**DOI:** 10.4102/ajlm.v3i2.221

**Published:** 2014-11-03

**Authors:** Laura T. Eno, Terence Asong, Elive Ngale, Beatrice Mangwa, Juliana Ndasi, Maurice Mouladje, Remmie Lekunze, Victor Mbome, Patrick Njukeng, Judith Shang

**Affiliations:** 1 US Centers for Disease Control and Prevention, Cameroon; 2 Global Health Systems Solution, Cameroon; 3 Buea Regional Hospital, Cameroon; 4 Cameroon Development Corporation, Central Clinic, Tiko; 5 Regional Delegation for Health, Buea, Cameroon

## Abstract

**Background:**

Inspired by the transformation of the Regional Hospital Buea laboratory through implementation of the Strengthening Laboratory Management Toward Accreditation (SLMTA) programme, hospital management adapted the SLMTA toolkit to drive hospital-wide quality improvement.

**Objective:**

This paper describes changes in the hospital following the quality improvement activities in hygiene and sanitation, the outpatient waiting area and the surgical and maternity wards.

**Methods:**

In March 2011, hospital management established a quality improvement task force and created a hospital-wide quality improvement roadmap, following the SLMTA model. The roadmap comprised improvement projects, accountability plans, patient feedback forms and log books to track quality indicators including patient wait time, satisfaction level, infection rates, birth outcomes and hospital revenue.

**Results:**

There was steady improvement in service delivery during the 11 months after the introduction of the quality improvement initiatives: patient wait time at the reception was reduced from three hours to less than 30 minutes and patient satisfaction increased from 15% to 60%. Treatment protocols were developed and documented for various units, infrastructure and workflow processes were improved and there was increased staff awareness of the importance of providing quality services. Maternal infection rates dropped from 3% to 0.5% and stillbirths from 5% to < 1%. The number of patients increased as a result of improved services, leading to a 25% increase in hospital revenue.

**Conclusion:**

The SLMTA programme was adapted successfully to meet the needs of the entire hospital. Such a programme has the potential to impact positively on hospital quality systems; consideration should be made for development of a formal SLMTA-like programme for hospital quality improvement.

## Introduction

High-quality healthcare service delivery is essential for improving patient outcomes and reducing mortality. Despite advances in expanding and strengthening public health services in developing countries, many hospitals continue to struggle to maintain quality systems.^1,2^ In order to meet accreditation standards that have been set forth to ensure uniform healthcare, comprehensive quality management systems (QMS) must be established throughout the hospital system.^3,4^ That being said, no hospital QMS has previously been piloted or implemented in Cameroon.

In the clinical laboratory setting, however, a proven quality improvement tool already exists, namely, the Strengthening Laboratory Management Toward Accreditation (SLMTA) programme. SLMTA is a competency-based management training programme designed to achieve immediate and measurable improvements in laboratory quality systems.^5^ Launched in Kigali in 2009, SLMTA has spread rapidly and, by the end of 2013, had been implemented in 617 laboratories in 47 countries.^6^ Practical approaches to addressing daily challenges, task-based methods and hands-on learning on management practices are amongst the innovative features of the SLMTA curriculum. Progress is measured using the World Health Organization Regional Office for Africa’s (WHO AFRO) Stepwise Laboratory Quality Improvement Process Towards Accreditation (SLIPTA) checklist,^7^ which is divided into 12 sections corresponding to the Quality System Essentials.^8^ Resulting audit percentage scores determine the quality ratings on a zero- (< 55%) to five- (≥ 95%) star scale.

The laboratory at Regional Hospital Buea (RHB) was one of five pilot laboratories selected by the Ministry of Health (MOH) to implement the SLMTA programme in Cameroon. A baseline audit of the RHB laboratory was performed in November 2009 by a WHO AFRO-trained local auditor. Three SLMTA training workshops were conducted, starting in October 2010 and interspersed with improvement projects and on-site mentoring. The exit audit was conducted in September 2012 and showed that RHB’s laboratory had made a marked improvement, rising from 18% (zero stars) at baseline to 67% (two stars) at the exit audit. Improvement was observed in 11 out of 12 Quality System Essential sections.

The successful implementation of SLMTA at RHB’s laboratory attracted the attention of hospital management, who were interested in building QMS throughout the facility. In March 2011, it was proposed that the SLMTA toolkit be tailored into an improvement programme for the entire hospital. This study describes how RHB adapted the SLMTA programme in order to strengthen hospital quality systems and to achieve measurable outcomes.

## Research methods and design

### Study area

RHB is located in the South West region of Cameroon, with a capacity of 120 beds and a workforce of 200 staff serving a population of 90 088. The hospital consults an average of approximately 30 000 patients annually, 40% of whom require laboratory services. In addition to laboratory analyses, the hospital's services include: medicine, paediatrics, obstetrics, gynaecology, surgery, dentistry, eye care, radiology and haemodialysis. RHB also serves as a teaching hospital for the faculty of Health Sciences at the University of Buea.

### Establishment of the Quality Improvement Task Force

Hospital leadership participated in one of the SLMTA workshops conducted for laboratory staff and proposed that other hospital units be engaged in similar quality improvement activities. To coordinate this effort, hospital management formed the Quality Improvement Task Force (QUITAF).

QUITAF was chaired by the hospital director, in collaboration with the general superintendent of the hospital as well as the laboratory director, who served as the quality assurance officer. Unit heads were appointed as quality officers, whilst clinicians were also engaged as members of QUITAF. QUITAF was mandated to develop a measurable hospital quality improvement plan so as to strengthen the delivery of hospital services, with the goal of improving patient care.

The responsibilities and accountability of QUITAF members were defined. Quality officers were primarily in charge of daily operations, implementation and maintenance of quality systems in their respective units. The quality assurance officer and the general superintendent provided high-level direction and oversaw the overall improvement activities, whilst technical guidance was provided by two SLMTA trainers and laboratory mentors from the US Centers for Disease Control and Prevention (CDC) Cameroon office. The hospital director reviewed and approved all technical and quality management plans developed by the staff and made financial decisions when necessary.

### Tailoring SLMTA for the entire hospital

Laboratory staff members who had been trained in SLMTA were assigned to work with QUITAF members to develop a hospital-wide quality improvement programme. Three training modules from the SLMTA toolkit (Cross-Cutting, Productivity Management and Work Area Management) were modified in order to address hospital-wide issues concerning turnaround time, floor plans and standardised procedures. Ten hospital staff members were trained for three days in April 2011 followed by mentorship provided by laboratory staff and the two CDC-Cameroon SLMTA trainers. Subsequent workshops were conducted in the various units based on specific gaps. Participants were encouraged to develop improvement projects in order to address major challenges in their units using minimal financial resources. Selection, implementation and results of improvement projects were discussed in the QUITAF meetings.

A quality improvement roadmap was created outlining action items for each key improvement area, with expected outcomes and deliverables (Table 1). This plan was reviewed during weekly team meetings in order to assess progress made, address key challenges and capture lessons learnt. The almoner team, which is responsible for all information concerning the various hospital units, services available and amounts charged per service delivery, disseminated pertinent information on the quality improvement plan to all.

**TABLE 1:** Quality improvement roadmap for Regional Hospital Buea.

Steps	Action items	Outcomes and/or Products
Create a pilot plan for quality improvement	Conduct site visits and hold consultative meetings	Pilot plan adopted
Conduct baseline assessment	Design patient feedback forms and distribute them to units involved for completion	Baseline results analysed
Train hospital staff, select improvement projects	Conduct training on four activities from SLMTA’s Cross-cutting module: Process Mapping, Managing Performance – The Balanced Scorecard, Planning Improvement Projects and Reporting Improvement Projects	10 staff members trained; improvement projects selected
Pilot the quality improvement plan	Conduct site assessment	Performance reports from the various hospital units reviewed
Modify the plan based on pilot results	Hold consultation meetings with relevant unit heads; conduct site visits; provide technical assistance	Quality improvement plan adopted
Implement the modified plan	Conduct mid-term review of pilot-phase data; host QUITAF meeting	Interim reports reviewed
Review outpatient feedback	Request patients provide feedback in suggestion boxes after each visit to the hospital	Feedback data analysed
Review and modify the plan if necessary before full hospital scale-up	Hold QUITAF meetings; conduct site visits	Report and recommendations amended

SLMTA, Strengthening Laboratory Management Toward Accreditation; QUITAF, Quality Improvement Task Force.

For evaluation purposes, baseline data for the number of patients served per month and patient wait times were established using past records from the patient log books and direct observation. Indicators were measured non-systematically using logs and reports from key informants. Maximum patient wait times were estimated by scanning log books in order to determine the total time from arrival of the patient in the outpatient reception area to when they left the room (patient wait time at reception) or when they left the laboratory with their results (overall patient wait time). The proportion of public toilets that were functional in the facility was assessed as a measure of hospital hygiene and sanitation. The infection rate as a result of poor hygiene was estimated by the theatre nurse, who reviewed admission records in the surgical ward before and after the improvement project. The overall infection rate was counted from the patient records; the number of infections related to poor sterilisation was estimated before and after program improvements. Stillborn and maternal infection rates were estimated by the midwife of the maternity ward using birth records. The hospital director provided an estimate of the number of patients served annually and the hospital revenue; he also initiated a staff survey to assess their awareness of quality improvement activities. Patients were asked to provide feedback on service quality. One thousand (500 each in March and September 2011) patient feedback forms were made available with suggestion boxes; patient satisfaction was estimated based on the proportion of returned forms with positive comments.

## Results

Patient wait time at reception decreased from three hours to less than half an hour (Table 2) and total patient wait time decreased from greater than three days to less than one day. Staff awareness of quality improvement programmes in the hospital increased from 10% to 75%. Overall patient satisfaction increased from 15% to 60%. The percentage of patients who said they would return to the RHB for consultation rose from 60% at baseline to 90% six months later; and 85% said they would recommend the hospital to their friends or relatives. The proportion of functioning public toilets increased from 10% to 75%.

**TABLE 2:** Improvement projects at the Regional Hospital Buea selected by each unit head in collaboration with the entire Quality Improvement Task Force (QUITAF maternal) team from April 2011 to February 2012.

Hospital unit	Improvement project goals	Responsible staff member	Data source	Comparison timeframes	Key outcome
Outpatient department	Reduce patient wait time in the reception room	Nurse	Patient registers during programme	March 2011 versus May 2011	Maximum wait time at reception decreased from > 3 hours to < 30 minutes
Maternity	Reduce still births and maternal infections by increasing use of the pathogram to record patients in labour	Midwife	Birth registers and chart review	January-March 2011 versus April-June 2011	Reduction in stillbirths related to poor follow-up from 5% to < 1%; infection rate dropped from 3% to 0.5%
Surgical ward/Theatre	Create a window between the sterilisation unit and theatre to reduce operation -related infections	State registered nurse	Observation and chart review	January-March 2011 versus April-June 2011	Reduced post-operation infections from five per month (out of 83 average total) to < 1 per month (out of 102 average total)
Hygiene and sanitation	Reduce litter, post signage and educate patients to keep hospital environment and toilet facilities clean	Janitor	Observation	March 2011 versus April 2011	Functioning public toilets increased from 10% to 75%
Laboratory	Draft clinician handbook to improve clinician understanding of laboratory services	Laboratory technologist	Observation and interview	June 2011	First draft of handbook produced
Staff	Conduct surveys to assess staff awareness of quality improvement activities	Hospital director	Structured questionnaire	March to December 2011	Staff awareness increased from 10% to 75%
Patients	Place suggestion boxes at all hospital units to identify issues and improve patient satisfaction	QUITAF quality officer	Returned suggestion slips (a total of 513 questionnaires returned out of 1000)	March 2011 versus September 2011	Patient satisfaction increased from 15% to 60%
Hospital finance and administrative department	Proper documentation to monitor the routine control of cash records	Accounts department/Director of hospital	Accounting records	February 2011 versus February 2012	Hospital revenue increased from 80 000 000 FCFA to 100 000 000 FCFA

QUITAF, Quality Improvement Task Force; FCFA, CFA Franc.

Other key indicators also showed marked progress: the maternal infection rate dropped from 3% to 0.5%; the number of stillbirths decreased from 5% to < 1%; and the number of patients served grew and hospital revenue increased by 25%. To improve sterilisation practices, a window was introduced between the sterilisation room and the preparation room in the theatre, minimising the exposure of surgical supplies to potential contamination by transporting them through the crowded hospital corridors. New systems were developed such as: a calendar for sterilisation of equipment; a log book for recording and counting sterilised forceps and disposable gowns; and placement of additional dust bins around the hospital. Signage was posted to indicate the location of units and to encourage proper hygiene habits within the hospital. Sessions were conducted to educate patients on proper use of the public toilets. These changes led to less foot traffic and increased cleanliness in the overall hospital environment.

## Discussion

Hospital management at RHB successfully adapted the SLMTA toolkit for a multi-disciplinary hospital-wide improvement programme. The QUITAF facilitated team work and held the units accountable for quality. Increased collaboration between clinicians and laboratory staff ensured that quality improvement efforts were patient-centred. Effective communication ensured that the entire hospital was aware of the changes and helped to foster a culture of quality. During QUITAF meetings, experiences and challenges were shared, leading to positive changes such as timeliness and consistency in the reporting of results. The implementation of a hospital-wide quality improvement plan allowed hospital management to evaluate its staff’s strengths, establish procedures and communicate more effectively.

The quality change implemented in the entire hospital and advances in hospital processes led to critical improvements in patient care, as reported in the reduction of wait times, infections and stillbirths. The public took notice; as patient satisfaction increased, so did the number of patients and hospital revenue.

Implementation of hospital-wide improvements in conjunction with SLMTA further enhanced the laboratory’s quality strengthening efforts. For example, the presence of laboratory staff during clinical rounds allowed critical issues to be discussed in real time. Clinicians began to better understand laboratory delays and hospital staff expressed increased confidence in relying on the hospital laboratory for testing, rather than on privately-owned facilities. In August 2014, an official WHO AFRO SLIPTA audit was conducted in the RHB laboratory by the African Society for Laboratory Medicine. The laboratory earned two SLIPTA stars (with an audit score of 69%), indicating that it is well on its way to developing an effective QMS. The audit report highlighted the complementary role played by QUITAF in supporting both laboratory and hospital quality improvement.^9^ Through QUITAF, the entire hospital management and staff were mobilised to support and contribute to sustaining the quality culture.

Following the implementation of the quality improvement programme, hospital management is now working to develop targets for the annual number of patients served, minimum revenue expected, maximum number of deaths due to poor practices and maximum number of customer complaints.

### Limitations of the study

This assessment is subject to several limitations. Modifying the SLMTA toolkit for the entire hospital was not always easy, as tools to measure improvement in non-laboratory settings were lacking. Patient feedback forms were created and, whilst written comments on the forms were useful, they were not easily quantifiable; more thorough qualitative analysis of this feedback is warranted. Most critically, many of the indicators reported were not observed directly or collected systematically, but were based on expert report and retrospective review of log books. Furthermore, because of logistical barriers, some variables were not measured before implementation, limiting our conclusions. The data presented here represent convenient, readily-available programmatic data, using pre-existing sources or those developed during the quality improvement initiative itself. Whilst results are encouraging, validation in other facilities using thorough scientific techniques is needed in order to draw solid conclusions.

### Conclusion

Modification of the SLMTA programme for the wider hospital setting allowed hospital management and staff to identify gaps and develop a plan to address them. The director of the RHB testified that, ‘[*q*]uality can be improved by making changes within the healthcare system without necessarily increasing resources’ and also that:

‘SLMTA is an invaluable tool for every lab director, every hospital manager and health policy maker because of its value in ensuring quality improvement within the laboratory and its potential in contributing to strengthening the entire health system at little or no cost.’

Expressing a similar opinion, the Ministry of Health and Social Services (MoHSS) laboratory director of Namibia said:

‘As quality improvements become institutionalised in hospital laboratories, it is becoming evident that entire hospital systems are in dire need of similar quality improvement programmes. The Namibia MoHSS calls on international agencies to develop and adapt programmes such as SLIPTA and SLMTA for use throughout hospital systems so as to ensure continuous quality patient care.’^10^

Given the positive experience at RHB, we second this call and recommend that a hands-on competency-based curriculum be developed fully in order to support hospital quality management, extending the Strengthening *Laboratory* Management Toward Accreditation programme into one for Strengthening *Hospital* Management Toward Accreditation.
